# Addressing Challenges in Wildlife Rehabilitation: Antimicrobial-Resistant Bacteria from Wounds and Fractures in Wild Birds

**DOI:** 10.3390/ani14081151

**Published:** 2024-04-10

**Authors:** Esther Sánchez-Ortiz, María del Mar Blanco Gutiérrez, Cristina Calvo-Fernandez, Aida Mencía-Gutiérrez, Natalia Pastor Tiburón, Alberto Alvarado Piqueras, Alba Pablos-Tanarro, Bárbara Martín-Maldonado

**Affiliations:** 1 Departamento de Sanidad Animal, Facultad de Veterinaria, Universidad Complutense, Avenida de Puerta de Hierro s/n, 28040 Madrid, Spain; estherortiiz@gmail.com (E.S.-O.); mmblanco@ucm.es (M.d.M.B.G.); 2 Grupo de Rehabilitación de la Fauna Autóctona y su Hábitat, Calle Monte del Pilar s/n, 28220 Majadahonda, Spain; aidamengu@gmail.com (A.M.-G.); natalia@grefa.org (N.P.T.); alberto@grefa.org (A.A.P.); 3 Research Group for Food Microbiology and Hygiene, National Food Institute, Technical University of Denmark, Henrik Dams Allé, 204, 2800 Kongens Lyngby, Denmark; ccfe@food.dtu.dk; 4 Research Group for Foodborne Pathogens and Epidemiology, National Food Institute, Technical University of Denmark, Henrik Dams Allé, 204, 2800 Kongens Lyngby, Denmark; 5 Departamento de Veterinaria, Facultad de Ciencias Biomédicas y de la Salud, Universidad Europea de Madrid, Calle Tajo s/n, 28760 Villaviciosa de Odón, Spain; alba.pablos@universidadeuropea.es

**Keywords:** *Staphylococcus*, *S. aureus*, *S. lentus*, AMR, multidrug resistance, clindamycin, avian medicine

## Abstract

**Simple Summary:**

Wildlife rescue centers frequently admit animals with injuries and bone fractures. Open fractures are common in birds due to their anatomy, and this can lead to complications like osteomyelitis, which implies a serious bone infection and often necrosis, or death of the affected bone tissue. Antibiotic therapy is crucial, but the rise in antimicrobial-resistant isolates in wildlife raises concerns about treatment efficacy. A study focused on isolating, identifying, and assessing antimicrobial resistance in bacteria from wounds and fractures in wild birds. Among 36 isolates, *Staphylococcus* spp. dominated (63.8%), with 82.6% exhibiting antimicrobial resistance, particularly to clindamycin, an antimicrobial key in the treatment of infected bone fractures. This escalating resistance poses a dual threat to wildlife—therapeutic failure and the spread of resistant bacteria in ecosystems.

**Abstract:**

Injuries and bone fractures are the most frequent causes of admission at wildlife rescue centers. Wild birds are more susceptible to open fractures due to their anatomical structure, which can lead to osteomyelitis and necrosis. Antibiotic therapy in these cases is indispensable, but the increase of antimicrobial-resistant isolates in wildlife has become a significant concern in recent years. In this context, the likelihood of antibiotic failure and death of animals with infectious issues is high. This study aimed to isolate, identify, and assess the antimicrobial resistance pattern of bacteria in wounds and open fractures in wild birds. To this end, injured birds admitted to a wildlife rescue center were sampled, and bacterial isolation and identification were performed. Then, antimicrobial susceptibility testing was assessed according to the disk diffusion method. In total, 36 isolates were obtained from 26 different birds. The genera detected were *Staphylococcus* spp. (63.8%), *Escherichia* (13.9%), *Bacillus* (11.1%), *Streptococcus* (8.3%), and *Micrococcus* (2.8%). Among *Staphylococcus* isolates, *S. lentus* and *S. aureus* were the most frequent species. Antimicrobial resistance was detected in 82.6% of the isolates, among which clindamycin resistance stood out, and 31.6% of resistant isolates were considered multidrug-resistant. Results from this study highlight the escalating scope of antimicrobial resistance in wildlife. This level of resistance poses a dual concern for wildlife: firstly, the risk of therapeutic failure in species of significant environmental value, and, secondly, the circulation of resistant bacteria in ecosystems.

## 1. Introduction

According to the International Union for Conservation of Nature (IUCN), 159 bird species have become extinct during the last 20 years, and now 12% of the described bird species are threatened [1]. Specifically in Europe, bird populations have declined by 600 million individuals since 1980 [2]. As species abundance has been described as a synonym of a healthy ecosystem, biodiversity conservation becomes key to ensuring global health for humans, animals, and the wild [3]. In this context, the role of wildlife rescue centers (WRCs) is essential as an ex situ action to ensure animal welfare, treating and caring for injured, sick, and stray native animals [4]. However, despite WRC efforts, the release rate ranges between 27% and 56% for wildlife admitted to WRCs, depending on the animal class and the region [5,6,7,8].

Among the causes of admission to a WRC, those linked to human activity, such as electrocutions, car or window collisions, or gunshots, are the most frequent [9,10]. In contrast, injuries due to other animals’ attacks are not so common but still are represented among traumatic causes of admission. Due to the anatomical adaptations of the bones for flight, birds are more prone to suffer open fractures with bone exposure [11,12]. In those species, the trauma category represents around 30–65% of the total admissions, and the prevalence of injuries and bone fractures in wild birds admitted to a WRC are, therefore, higher and the most common cause of morbidity and mortality [4,5,6,7]. A high percentage of wild birds admitted due to a traumatic cause, involving injuries and/or fractures, had a negative outcome, either by death or euthanasia [6]. This, coupled with the time elapsed from the trauma until the animal’s capture and arrival at the WRC, increases the risk of infection that can lead to osteomyelitis and necrosis [5,13,14,15]. The majority of avian wounds are older than 8 h and/or contaminated by the time they present for treatment [16]. The anatomical point of fracture, the extension of soft-tissue lesions, the infection and osteomyelitis, or the bone and soft-tissue necrosis are some of the criteria that help veterinarians assess euthanasia [6,17]. Unlike in mammals, osteomyelitis in birds is typically not systemic unless it involves a pneumatic bone, like the humerus or femur. This communication between the fracture and infection focus and the respiratory system can lead to fatal pneumonia [18].

The bacterial genera most frequently involved in wounds and open fractures in wild birds are mainly *Staphylococcus, Enterococcus*, *Bacillus*, *Aeromonas*, and other bacteria from the Enterobacteriaceae family, such as *Escherichia*, *Enterobacter*, *Shigella*, and *Proteus* [13,14]. *Staphylococcus* spp. is a ubiquitous bacterium in the normal microbiota of humans and animals, with the ability to cause a broad spectrum of infections [19]. Concretely, *Staphylococcus aureus* is one of the seven ESKAPE micro-organisms producing resistant infections in hospitals worldwide. The name of this group of highly virulent and antimicrobial-resistant bacteria (ARB) is the acronym of their seven scientific names: *Enterococcus faecium*, *Staphylococcus aureus*, *Klebsiella pneumoniae*, *Acinetobacter baumannii*, *Pseudomonas aeruginosa*, *Enterobacter* spp., and *Escherichia coli* [20]. The infection with *S. aureus* is the most common and can exacerbate osteomyelitis development through exfoliative and pore-forming toxins and superantigens that promote osteoclastogenesis and bone resorption [18]. Moreover, *S. aureus* has been described as one of the species with the highest rate of antimicrobial resistance (AMR) and resistant strains, which have been associated with more than 700,000 deaths worldwide in 2019 [21].

Despite the fact that wild bird rehabilitation from trauma usually entails antibiotic therapy, it must be conducted in a way so as to avoid the development and increase of new resistant bacteria. Nowadays, antimicrobial resistance (AMR) is one of the biggest health issues in the world, causing millions of human deaths yearly. Treating these bacterial infections has become arduous due to increased antimicrobial resistance [21]. In recent years, many authors have published the isolation of ARBs from wild birds, even though wildlife should not have direct contact with clinical settings [14,19,22,23]. This is due to the infinite interactions between humans and wildlife, direct or indirect, and the ecosystem’s pollution with antimicrobial residues [24,25]. Moreover, migratory birds have been suggested to have a higher probability of carrying ARBs in their microbiome and disseminating them through different regions due to the vast territory they cover [25,26]. Therefore, wild birds are crucial in disseminating ARB between environments and habitats [26].

This study aimed to isolate and characterize the bacteria in wounds and open fractures in wild birds admitted to a WRC from Spain. As a secondary objective, antimicrobial susceptibility tests were conducted to refine treatment protocols for wild birds accurately.

## 2. Materials and Methods

### 2.1. Sample Collection

From November 2018 to May 2020, wild birds admitted because of trauma at the WRC managed by Grupo de Rehabilitación para la Fauna Autóctona y su Hábitat (GREFA) were examined and sampled if their health status allowed it. Wound or open fracture samples were collected for microbiological diagnosis and antimicrobial susceptibility test with a sterile swab during their first examination. Samples were preserved in a liquid nutrient transport medium with fructose-1,6-bisphosphatase (FBP) (Oxoid^®^, Basingstoke, UK) and 0.5% active charcoal (Sigma-Aldrich^®^, Saint Louis, MO, USA) [27]. Then, samples were frozen at −20 °C until analysis at the laboratory. Handling procedures complied with Spanish legislation (Royal Decree 53/2013) [28]. Ethical review and approval were waived for this study because of the standard protocol for the sanitary status analysis of animals admitted to the GREFA Wildlife Hospital. Therefore, no extra handling of the animals was necessary to collect the samples, and no extra samples were collected outside the hospital’s standard workflow protocol.

### 2.2. Bacterial Culture and Isolation

At the laboratory, samples were unfrozen at room temperature (20–15 °C). After homogenization in a vortex, 100 µL of each sample were collected and transferred onto two agar media: Columbia Agar with 5% sheep blood and MacConkey (Oxoid Ltd.^®^, Basingstoke, UK). The blood agar culture was performed in aerobic and anaerobic conditions with AnaeroGen^®^ (Thermofisher Scientific^®^, Waltham, MA, USA). All plates were incubated at 37 ± 1 °C for 24 h. Then, the macroscopic characteristics of different colonies formed on each plate were observed (size, shape, and color of the colonies; presence of alpha- or beta-hemolysis or no hemolysis on blood agar cultures; and whether there was lactose fermentation or not on MacConkey cultures) and used to sort them out. Plates without growth in the first 24 h were kept for another 24 h under the same conditions before confirming negative growth. Then, a single colony of each morphology present was selected from each plate with growth and stroked on Columbia Base (Oxoid Ltd.^®^, Basingstoke, UK) to obtain a monoclonal culture after 24 h of incubation at 37 ± 1 °C. A sample of each monoclonal culture was preserved in cryovials with nutritive broth and glycerol (80–20%, respectively) at −80 °C for further analysis.

### 2.3. Bacterial Identification

Bacterial identifications were performed by microscopical morphology, shape, and staining characteristics with Gram stain (Panreac AppliChem^®^, Darmstadt, Germany) and classical biochemical tests, including catalase, potassium hydroxide (KOH) (MERCK^®^, Darmstadt, Germany), and oxidase (MAST^®^ ID, Merseyside, UK) [29]. Additionally, isolates whose characteristics were compatible with *Staphylococcus* spp. were identified at the species level using Analytical Profile Index (API) STAPH multisubstrate galleries (bioMérieux^®^, Marcy l’Etoile, France). In the same way, isolates compatible with *E. coli* were confirmed with API 20E multisubstrate galleries (bioMérieux^®^, Marcy l’Etoile, France). Positive controls of *S. aureus* (ATCC 25923) and *E. coli* (ATCC 4157) were included in the analysis with API STAPH and API 20E galleries, respectively.

### 2.4. Antimicrobial Susceptibility Test

Antimicrobial susceptibility test (AST) was performed according to the disk diffusion or Kirby–Bauer method and the European Committee on Antimicrobial Susceptibility Testing (EUCAST) guidelines [30]. Isolates were retrieved from cryovials and plated onto Columbia Agar supplemented with 5% sheep blood (Becton Dickinson GmbH, Heidelberg, Germany). A suspension of the inoculum was prepared in a sterile 0.8% saline solution to achieve a turbidity equivalent to 0.5 McFarland. Subsequently, the inoculum was transferred onto Mueller–Hinton agar (Becton, Dickinson GmbH, Heidelberg, Germany), and antimicrobial disks were placed on the surface. The selection of antimicrobials for the susceptibility test was based on the panel of antimicrobials routinely used in treating wild birds at GREFA Wildlife Hospital. According to EUCAST recommendations, the antimicrobial susceptibility analysis of staphylococci includes chloramphenicol, clindamycin, erythromycin, tetracycline, linezolid, and tedizolid [31]. However, the latter two are scarcely used in veterinary medicine, especially in wildlife, so they were not included in the test. In exchange, ampicillin, ciprofloxacin, and trimethoprim-sulfamethoxazole were added, as they are commonly used in wildlife clinical practice. In total, susceptibility to seven groups of antibiotics was analyzed: penicillins, quinolones, sulphonamides, macrolides, lincosamides, amphenicols, and tetracyclines (Table 1). After 24 h at 37 ± 1 °C, susceptibility or resistance was determined by growth inhibition diameter regarding standardized breakpoint tables from EUCAST [31]. For the antimicrobial without cut-off values in EUCAST tables (chloramphenicol), the breakpoint was established following the Clinical and Laboratory Standards Institute (CLSI) guidelines [32]. Multidrug resistance (MDR) was considered when the isolate was non-susceptible to at least one antimicrobial agent in three or more different classes of antimicrobial [33].

## 3. Results

Samples of 26 individuals belonging to 11 different species of birds were analyzed: black kite, booted eagle, Eurasian eagle-owl, Tawny owl, little owl, mallard, white stork, grey heron, lesser black-backed gull, common blackbird, and rock dove (Table 2).

### 3.1. Bacterial Isolation and Identification

**Figure 1:**
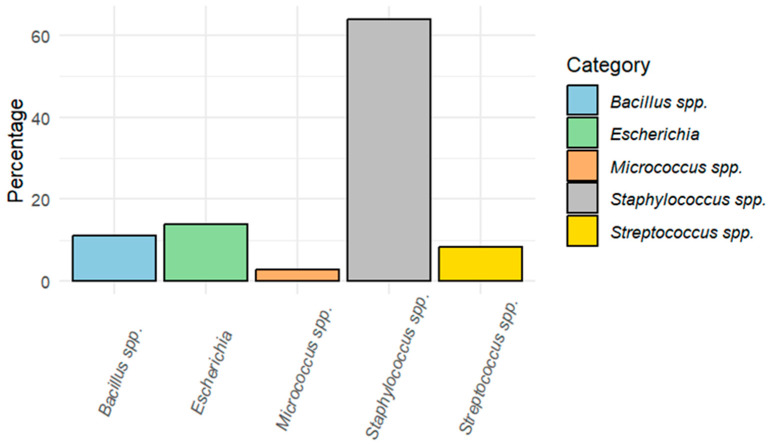
The percentage of the different bacterial genera was identified after basic biochemical tests and Gram staining.

**Figure 2:**
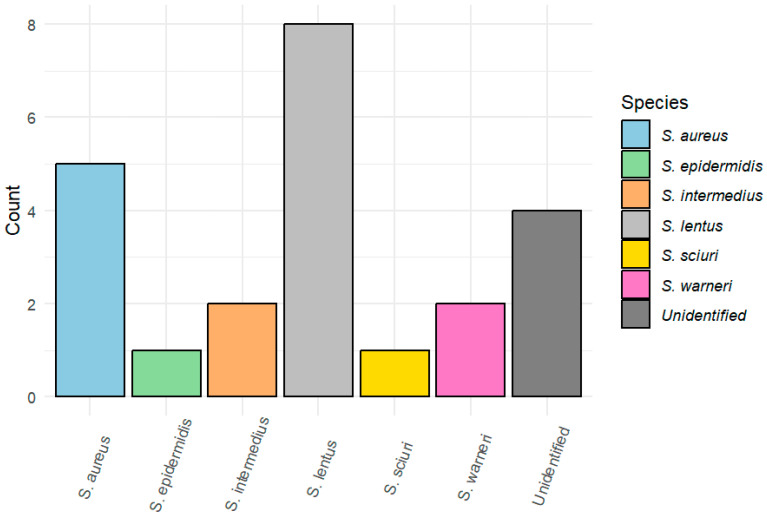
*Staphylococcus* species identified using API STAPH^®^(bioMérieux^®^, Marcy l’Etoile, France).

Positive bacterial growth was obtained in 14 of the 26 including birds (53.8%), while the other 12 birds had no bacterial growth in any of the media used. In total, 36 different colonies were isolated. After basic biochemical tests and Gram staining, they could be classified at the genus level: 63.8% (23/36) *Staphylococcus* spp., 13.9% (5/36) *Escherichia*, 11.1% (4/36) *Bacillus* spp., 8.3% (3/36) *Streptococcus* spp., and 2.8% (1/36) *Micrococcus* spp. (Figure 1). Moreover, *Staphylococcus* isolates could be identified at the species level using API STAPH^®^ galleries, with reliability ≥ 90%: *S. lentus* (34.8%, 8/23), *S. aureus* (21.7%, 5/23), *S. warneri* (8.7%, 2/23), *S. intermedius* (8.7%, 2/23), *S. epidermidis* (4.3%, 1/23), and *S. sciuri* (4.3%, 1/23) (Figure 2). *S. lentus* was the species most isolated from open-wound and fracture samples. The staphylococci species could not be identified in four isolates (17.3%) (Table 2). All the *Escherichia* isolates belonged to the species *E. coli*.

### 3.2. Antimicrobial Susceptibility Results

As *Staphylococcus* was the genus most frequent and because it has been considered a sentinel for AMR surveillance, AST was performed only with *Staphylococcus* isolates. Of the 23 staphylococci isolates, 82.6% were resistant to at least one antimicrobial (19/23). Six isolates were susceptible to all the antimicrobials tested: two from Eurasian eagle-owls, two from white storks, one from a black kite, and the last one from a booted eagle. Still, none showed efficacy in all the isolates. The highest percentage of resistance found was to clindamycin (CMN) (52.2%, 12/23), followed in decreasing order by ampicillin (AMP), erythromycin (ERY), tetracycline (TET), and trimethoprim-sulfamethoxazole (SXT), ciprofloxacin (CIP), and, finally, chloramphenicol (CHL) (Table 3).

Overall, 31.6% of resistant isolates (6/19) were considered MDR since they were resistant to at least three different antimicrobial classes, half of them (50%, 3/6) to four antimicrobial classes. All MDR isolates were resistant to CMN combined with different antimicrobials, mainly cephalosporins, penicillins, and/or erythromycin. S. lentus was the species with a higher rate of MDR. Regarding bird species, the grey heron has the highest rate of MDR staphylococci, followed by the white stork. In contrast, lesser black-backed gulls and Eurasian eagle-owls were the only species without MDR strains.

## 4. Discussion

The role of WRC in treating injured wildlife could be key for the restoration of endangered populations as an ex situ tool [4]. As one of the main causes of admission of wild birds is trauma, the management of injuries and fractures becomes essential for the successful in wildlife rehabilitation, even more so in wild birds due to their anatomical adaptations to flight [11]. However, there is a lack of studies about the distribution of bacteria in injuries and open fractures in wild birds, as well as their antimicrobial resistance patterns, which is of great importance in establishing the best treatment. Moreover, the presence of resistant bacteria in wild birds could represent a potential hazard to global health in the dissemination of resistant pathogens, many with zoonotic potential, such as *Escherichia coli*, *Salmonella enterica*, or *Staphylococcus aureus*. During their daily movements and migration, they can transport these resistant pathogens to new areas and spread them to the environment [14,25]. The present study assessed the bacterial genera most present in open fractures and wounds in wild birds admitted at a WRC.

In this work, the genus most frequently isolated was *Staphylococcus* (63.8%), followed by *Escherichia* (13.9%), *Bacillus* (11.1%), *Streptococcus* (8.3%), and *Micrococcus* (2.8%). These results contrast with those published by Gambino et al. [23], where the prevalence of *Staphylococcus* and *Streptococcus* were lower (11.4% and 3.1%, respectively), and no *Bacillus* were detected. *Staphylococcus* in birds was the most frequent genus in the samples analyzed and is highly relevant in human and veterinary medicine due to its ability to cause infections in the heart, bone, skin, and other tissues [34,35]. It is one of the genera with the highest resistance to antimicrobials, which makes its treatment extremely difficult, and *S. aureus* is considered one of the most lethal resistant bacteria worldwide, which globally had been indirectly associated with more than 4 million deaths in 2019 [21]. In addition, due to its ubiquity and easy cultivation, it has been used in numerous studies as a sentinel bacterium to study AMR trends [36,37]. The source of these bacteria colonizing injuries from wild birds might be related to scratches or bites from other animals, especially in urban wild birds. A mixed aerobic/anaerobic population is often isolated from infected bite wounds from small animals like felids and canids, which are also known to carry bacterial groups like *Staphylococcus* spp. and *Streptococcus* spp. on their gingival tissue and teeth. This is why antimicrobial therapy is always indicated in any bird attacked by other animals [38].

Within the genus *Staphylococcus*, the most frequently isolated species was *S. lentus* (34.8%) (proposed to be reclassified as *Mammallicoccus lentus* by Madhaiyan et al. [39]), which agrees with a study performed on nocturnal raptors where the prevalence of that species was 29.7% [19]. It has previously been identified in the trachea of healthy wild birds as a component of the respiratory microbiota and is also found on the skin of healthy pigeons [40,41]. Moreover, *S. lentus* has been isolated from the skin of people working with pigeons and described as a neglected pathogen for humans, but, to the best of our knowledge, there is no scientific report about zoonotic transmission [41,42]. In the present study, *S. lentus* was isolated from six individuals: three of them had open fractures of pneumatic bones (humerus and femur), so the presence of *S. lentus* in these fractures could be related to the respiratory microbiota. Instead, *S. aureus* was the second species most detected from open fractures and wounds; our detection rate is moderate (21.7%), according to Silva et al. [19]. The role of *S. aureus* as a zoonotic pathogen with high AMR rates is well-documented in both humans and animals, even in wild birds [19,21]. In a lower percentage, two isolates of *S. intermedius* and *S. warneri*, a species found in the feathers of migratory birds without associated symptoms, were obtained [43]. As a coagulase-positive *Staphylococcus* (CoPS), *S. intermedius* had an increased pathogenicity. On the contrary, *S. sciuri* is the most common coagulase-negative staphylococcal (CoNS) species in healthy wild animals, including birds, but it has a high zoonotic potential [40,43,44]. Now proposed also as *Mammallicoccus sciuri* [39], it was one of our study’s less dominant staphylococci species. These CoNS were considered less pathogenic than *S. aureus*, but recent studies have demonstrated an increasing clinical impact and can act as opportunistic pathogens [45,46]. Interestingly, a recent study on wild birds from Spain reported *S. sciuri* as the most frequent staphylococci species [40]. In another study, Sousa et al. [47] confirmed the presence of *S. sciuri* in rodents, rats, and squirrels, which can be part of the birds’ prey diet and could be a source of infection. Finally, only one isolate of *S. epidermidis* was obtained, which is considered an essential opportunistic species in human infectious diseases, and it has been described as part of the respiratory and digestive microbiota of birds in low concentration [40,47].

The antimicrobial susceptibility test of *Staphylococcus* spp. isolates showed a concerning proportion of AMR isolates (82.6%) from infected open fractures and wounds from wild birds. Even though ARB should be expected to be in a lower proportion in wild birds, some species, such as white storks (*Ciconia Ciconia*) or Columbiformes, are considered urban birds and have closer contact with human activities and garbage, increasing the risk of acquiring ARB [25,48]. Clindamycin was the antimicrobial with the highest rate of resistance (54.5%), which agrees with previous reports from wildlife [14,19,23,37,49]. Ruiz-Ripa et al. [40] detected 90% of CMN-resistant staphylococci, highlighting a potential conflict with veterinary clinical procedures as clindamycin is widely used, especially for pododermatitis and osteomyelitis treatment in wild birds [5,14]. In this context, a recent in-vitro experiment has demonstrated that lower doses of CMN can favor osteoblast proliferation and differentiation, as well as calcium deposition [50]. Beta-lactams are also common in both human and veterinary practice as the first choice, and resistance to this antimicrobial family is frequent in staphylococci isolated from wildlife [51]. Among penicillins, the rate of resistance to AMP was high (39.1%), which agrees with the data published by some authors [23,49]. This resistance against AMP has also been reported in livestock, companion animals, and humans in similar or higher proportions. This finding, added to the critical role of wildlife in disseminating and maintaining AMR in the ecosystems, confirms the connections between humans, animals, and the environment, and the need for a One Health approach to monitoring and curbing the dissemination of AMR [52,53]. While some authors recommend using clindamycin in osteomyelitis treatment, others suggest that cephalosporins such as ceftiofur or cefotaxime could give better outcomes [14]. However, third-, fourth-, and fifth-generation cephalosporins have been classified as the highest-priority critically important antimicrobials by the World Health Organization (WHO), so their use in veterinary practice should be limited [54]. Our results suggest that clindamycin is not the best option to treat open fractures or wounds infected by *Staphylococcus* spp. However, in our study, no cephalosporins were included, so it is not possible to assess the resistance of our strains to this class of antimicrobials. According to the published CLSI guidelines, resistance to some cephalosporins and other beta-lactam antimicrobials can be extrapolated from the result obtained from AST against oxacillin. Therefore, despite being an antimicrobial rarely used in veterinary medicine, the inclusion of oxacillin in the AST would have been very informative [32]. On the other hand, the absence of CHL-resistant isolates found in the present study contrasts with results obtained by Sousa et al. in a study of the characterization of staphylococci isolated from the nasal cavity in wild birds (71.3%) [47]. Resistance to ERY, CIP, TET, and SXT has also been observed in lower proportions in similar studies with wild birds [14,37,40,48,51].

From the 19 strains of resistant *Staphylococcus* spp., 6 were considered MDR (31.6%). This percentage is close to that reported in previous studies on wild birds, which showed percentages near to 50% of MDR [14,40]. In contrast, Fernandez-Fernandez et al. [37] confirmed the absence of MDR strains in 259 staphylococci isolates from the white stork respiratory system. MDR has been widely assessed in wildlife under a public-concern approach. However, it is important to highlight that the emergence of bacteria resistant to multiple antimicrobial classes could precipitate treatment failures in these animals, many of which play pivotal ecological roles.

Finally, to obtain more precise and statistically significant results, a larger number of cases is necessary. However, this study includes a small number of cases due to the complexity of obtaining complete clinical cases and collecting samples before any veterinary treatment, always prioritizing animal welfare when working with wildlife. Therefore, the results presented in this study are simply observational, and no statistical conclusions can be drawn from them. Further research is needed to evaluate and characterize the bacterial species that may be involved in infected wounds and open fractures in wild birds, including their resistance patterns.

## 5. Conclusions

In conclusion, the most frequent bacteria isolated from wounds and open fractures of wild birds in our study were *S. lentus*, followed by *S. aureus* and *E. coli*. Among staphylococci isolates, the antimicrobial resistance proportion was concerning (82.6%); mainly clindamycin, ampicillin, and erythromycin, and 31.6% of the resistant isolates were considered MDR. Although clindamycin benefits osteoblast proliferation, its administration should be avoided in infected open fracture treatment. Our study’s outcomes emphasize the alarming expansion of antimicrobial resistance in wildlife. This heightened resistance threatens therapeutic success in species of paramount environmental importance, intensifies the spread of ARB throughout ecosystems, and focuses attention on the urgent need to incorporate wildlife into surveillance protocols for antimicrobial resistance. Despite budget limitations linked to wildlife research, incorporating new molecular techniques such as sequencing in epidemiological studies would help us to understand whether the observed resistance mechanisms are acquired and share genomic structures with those identified in bacteria from other environments.

## Figures and Tables

**Table 1:** Details about antimicrobial disks used in the antimicrobial susceptibility test. Bio-Rad^®^, Hercules, CA, USA; Oxoid^®^, Basingstoke, United Kingdom.

Class of Antimicrobial	Antimicrobial	Code	Concentration (µg)	Supplier	Diameter Cut-Off (mm)
Penicillins	Ampicillin	AMP	10	Oxoid^®^	≤18
Quinolones	Ciprofloxacin	CIP	5	Oxoid^®^	≤17
Sulphonamides	Trimethoprim-sulfamethoxazole	SXT	25	Bio-Rad^®^	≤14
Macrolides	Erythromycin	ERY	15	Bio-Rad^®^	≤21
Lincosamides	Clindamycin	CMN	2	Bio-Rad^®^	≤22
Amphenicols	Chloramphenicol	CHL	30	Oxoid^®^	≤12
Tetracyclines	Tetracycline	TET	30	Oxoid^®^	≤22

**Table 2:** Taxonomic details about the individuals included in the study, population distribution, and bacteria isolated from the different lesions.

Order	Family	Species	Number of Birds	Lession	Bacteria Isolated
**Birds of prey**					
Accipitriformes	Accipitridae	Black kite (*Milvus migrans*)	6	Open fracture (*n* = 5)	*S. aureus*, *Bacillus* spp.
				Wound (*n* = 1)	*S. aureus*, *S. sciuri*, *S. warneri*
		Booted eagle (*Aquila pennata*)	3	Open fracture (*n* = 1)	Negative culture
				Wound (*n* = 2)	*S. lentus*, *Bacillus* spp., *E. coli*
Strigiformes	Strigidae	Eurasian eagle-owl (*Bubo bubo*)	1	Open fracture (*n* = 1)	*S. lentus*, *S. intermedius*, *E. coli*
		Tawny owl (*Strix aluco*)	2	Open fracture (*n* = 1)	Negative culture
				Wound (*n* = 1)	Negative culture
		Little owl (*Athene noctua*)	2	Open fracture (*n* = 2)	Negative culture
**Aquatic birds**					
Anseriformes	Anatidae	Mallard (*Anas platyrhynchos*)	2	Open fracture (*n* = 2)	Negative culture
Pelecaniformes	Ardeidae	Grey heron (*Ardea cinerea*)	1	Open fracture (*n* = 1)	*S. lentus*, *Micrococcus* spp., *E. coli*
Charadriiformes	Laridae	Lesser black-backed gull (*Larus fuscus*)	2	Open fracture (*n* = 1)	Negative culture
				Wound (*n* = 1)	*S. intermedius*
**Urban birds**					
Ciconiformes	Ciconidae	White stork (*Ciconia ciconia*)	5	Open fracture (*n* = 1)	*Staphylococcus* spp., *S. lentus*, *Streptococcus* spp., *E. coli*
				Wound (*n* = 4)	*S. lentus*, *S. epidemidis*, *S. warneri*, *Streptococcus* spp., *E. coli*
Columbiformes	Columbidae	Rock dove (*Streptotelia decaopto*)	1	Open fracture (*n* = 1)	Negative culture
Passeriformes	Turdidae	Common blackbird (*Turdus merula*)	1	Open fracture (*n* = 1)	Negative culture

**Table 3:** Antimicrobial resistance patterns among the staphylococci isolated obtained from wild bird’s wounds or open fractures. Resistant isolates: light grey.

Bird Species	*Staphylococcus* Species	ID	Zone Diameter ^a^ (mm)						
			AMP	CIP	ERY	CMN	CHL	TET	SXT
Black kite (*Milvus migrans*)	*S. aureus*	8	44	11	26	26	30	32	30
		9	45	11	25	27	32	32	30
		16	15	30	24	22	30	32	24
		18	15	30	26	26	32	32	28
	*S. sciuri*	17	27	31	28	18	30	37	26
	*S. warneri*	29	40	23	33	33	23	35	38
Booted eagle (*Aquila pennata*)	*S. lentus*	11	37	25	36	34	19	25	22
		13	11	30	14	0	25	18	25
Eurasian eagle-owl (*Bubo bubo*)	*S. intermedius*	1	29	42	27	28	34	23	32
	*S. lentus*	2	25	34	29	30	30	26	30
Grey heron (*Ardea cinerea*)	*S. lentus*	33	0	40	11	0	30	23	0
		35	0	38	10	0	30	25	0
Lesser black-backed gull (*Larus fuscus*)	*S. intermedius*	7	37	30	24	1	19	25	29
White stork (*Ciconia ciconia*)	*S. aureus*	20	20	35	0	31	30	43	34
	*S. epidermidis*	19	55	35	30	28	34	40	33
	*S. lentus*	3	17	40	10	0	30	25	26
		4	14	37	10	0	30	24	28
		25	18	34	30	36	26	23	0
	*S. warneri*	30	20	40	28	31	20	27	40
	*Staphylococcus* spp.	22	24	30	23	9	25	26	28
		24	22	22	24	7	22	28	32
		31	0	29	22	0	22	22	20
		32	25	25	21	0	27	22	26
Prevalence			39.1% (9/23)	8.7% (2/23)	30.4% (7/23)	52.2% (12/23)	0% (0/23)	13% (3/23)	13% (3/23)

^a^ Antimicrobials: AMP: ampicillin, CIP: ciprofloxacin, ERY: erythromycin, CMN: clindamycin, CHL: chloramphenicol, TET: tetracycline, SXT: trimethoprim-sulfamethoxazole.

## Data Availability

The data presented in this study are available on request from the corresponding author.
